# Western Diet Decreases Hepatic Drug Metabolism in Male LDLr^−/−^ApoB^100/100^ Mice

**DOI:** 10.1155/2023/5599789

**Published:** 2023-03-31

**Authors:** Markus Koponen, Jaana Rysä, Anna-Kaisa Ruotsalainen, Olli Kärkkäinen, Risto O. Juvonen

**Affiliations:** ^1^School of Pharmacy, Faculty of Health Sciences, University of Eastern Finland, Yliopistonranta 1, 70210 Kuopio, Finland; ^2^Finnish Safety and Chemicals Agency, PL 66 00521, Helsinki, Finland; ^3^A. I. Virtanen Institute for Molecular Sciences, University of Eastern Finland, Kuopio, Finland

## Abstract

Consumption of a Western diet is an important risk factor for several chronic diseases including nonalcoholic fatty liver disease (NAFLD), but its effect on the xenobiotic metabolizing enzyme activities in the liver has been studied incompletely. In this study, male LDLr^−/−^ApoB^100/100^ mice were fed with Western diet (WD) or a standard diet for five months to reveal the effects on drug metabolism such as cytochrome P450 (CYP) oxidation and conjugation activities in the liver. Hepatic steatosis, lobular inflammation, and early fibrosis were observed in WD fed mice, but not in chow diet control mice. When compared to the controls, the WD-fed mice had significantly decreased protein-normalized CYP probe activities of 7-ethoxyresorufinO-deethylation (52%), coumarin 7-hydroxylation (26%), 7-hydroxylation of 3-(3-fluoro-4-hydroxyphenyl)-6-methoxycoumarin (70%), 7-hydroxylation of 3-(4-trifluoromethoxyphenyl)-6-methoxycoumarin (78%), 7-hydroxylation of 3-(3-methoxyphenyl)coumarin (81%), and pentoxyresorufin O-depentylation (66%). Increased activity was seen significantly in sulfonation of 3-(4-methylphenyl)-7-hydroxycoumarin (289%) and cytosol catechol O-methyltranferase (COMT, 148%) in the WD group when compared to the controls. In conclusion, the WD-induced steatosis in male LDLr^−/−^ApoB^100/100^ mice was associated with decreased CYP oxidation reactions but had no clear effects on conjugation reactions of glucuronidation, sulfonation, and cytosolic catechol O-methylation. Consequently, the WD may decrease the metabolic elimination of drugs compared to healthier low-fat diets.

## 1. Introduction

Consumption of the so-called Western diet (WD) is a key risk factor for several chronic diseases such as type 2 diabetes, obesity, coronary heart diseases, and degenerative diseases [1]. The WD also promotes the development of several type of liver disorders from the benign form of nonalcoholic fatty liver disease (NAFLD) to the more severe nonalcoholic steatohepatitis (NASH) with or without fibrosis [2]. In NAFLD, an excessive amount of triglycerides accumulate in hepatocytes, leading to hepatic steatosis and chronic inflammation, degeneration of hepatocytes, necrosis, and the formation of fibrosis [2, 3].

The liver is the most important organ for the metabolism of xenobiotics, e.g., drugs, whose effects and effective time are greatly affected by their metabolism in the liver [4, 5]. The metabolism of drugs and other xenobiotics occurs in oxidation, reduction, hydrolysis, and conjugation reactions catalyzed by drug-metabolizing enzymes. The most important group of the drug-metabolizing enzymes are cytochrome P450s (CYPs), which are responsible for the metabolism of about 75% of clinically used drugs [6, 7]. These reactions are catalyzed mainly by five liver CYP enzymes, i.e., CYP1A2, CYP2C9, CYP2C19, CYP2D6, and CYP3A4. Some other important xenobiotic-metabolizing enzymes are UDP-glucuronosyltransferases (UGTs) and sulfotransferases (SULTs), which can conjugate the hydroxylated metabolites of CYP oxidation reactions. Both the types of drug-metabolizing enzymes and their amounts in the liver determine the rate and routes of metabolism and thus the elimination of xenobiotics. The same types of reactions take place for xenobiotics in humans and other mammalian species by the corresponding drug-metabolizing enzymes, although there are variations of levels, and the primary structures of the metabolizing enzymes often cause differences in rates and pathways between species [8].

While the basic level of drug metabolism and enzymes involved such as CYPs, UGTs, and SULTs is determined by the genome and age of the individual, they are also affected by several exogenous factors such as health-disease status, the presence of obesity, the diet, as well as exposure to inhibiting or inducing substances [4, 5]. In obese individuals, CYP2E1, 2D6, 3A4, and 3A5 metabolized drugs are eliminated faster [9–11]. Conversely, CYP1A2 has been observed to be suppressed in obese and NAFLD patients, and finally those drugs which are dependent on CYP1A2 oxidation such as theophylline are eliminated slowly in these individuals [12]. In animal models, CYP2C and CYP2E have been associated with faster xenobiotic metabolism in obese animals, whereas other liver CYP displayed signs of decreased metabolism [13]. The changes in conjugation reactions in NAFLD patients seem to be dependent on the type of conjugation, e.g., activity of SULT2A1 is decreased [14], whereas changes in glucuronidation rates have been inconsistent [14–16].

Mice with genetic manipulations such as leptin deficiency (ob/ob) [17], dysfunctional leptin receptor (db/db), or disturbances in lipid metabolism such as deficiency in low-density lipoprotein receptor (LDLr^−/−^) and expression of apolipoproteins or their combinations are used as models to mimic metabolic disorders [18, 19]. LDLr-deficient mice expressing only apolipoprotein B100 (LDLr^−/−^ApoB^100/100^) are a model in which spontaneous mild hypercholesterolemia and hypertriglyceridemia exist in conjunction with an increased plasma level of LDL [19, 20]. Therefore, we anticipated that these mice would be likely to develop moderate hepatic steatosis after prolonged consumption of a WD, and this would be accompanied by obesity.

The aim of the study was to investigate whether consumption of a WD would affect the xenobiotic-metabolizing enzyme activities in the liver of male LDLr^−/−^ApoB^100/100^ mice. Therefore, probe activities of CYP, UGT, SULT, and catechol O-methyltransferase (COMT) of microsomal or cytosol samples were determined from the liver samples of male LDLr^−/−^ApoB^100/100^ mice fed with the WD or the standard diet for five months.

## 2. Materials and Methods

### 2.1. Chemicals

Alamethicin, coumarin, 7-hydroxycoumarin, 7-hydroxy-4-trifluoromethylcoumarin (HFC), 7-ethoxyresorufin, 7-pentoxyresorufin, esculetin (98% purity), scopoletin (99% purity), PAPS, S-adenosylmethionine (SAM), trichloroacetic acid (TCA), UDP-glucuronic acid sodium salt (UDPGA), MnCl_2_, MgCl_2_, isocitric acid, isocitric acid dehydrogenase, and resorufin were purchased from Sigma-Aldrich (Steinheim, Germany). Glycin and KCl were from J.T. Baker (Deventer, The Netherlands). Albumin Bovine fraction V was purchased from MP Biomedicals, LLC. Bio-Rad was from Bio-Rad Laboratories, Inc. NADP^+^ was from Roche Diagnostics (Mannheim, Germany). NADPH regenerating system contained 1.12 mM NADP^+^, 12.5 mM MgCl_2_, 12.5 mM MnCl_2_, 16.8 mM isocitric acid, 0.056 mM KCl, and 15 U isocitric acid dehydrogenase in a 188 mM Tris-HCl buffer, pH 7.4. Water was deionized by Milli-Q gradient A10.

Coumarin derivatives were synthesized using Perkin–Oglialor condensation reactions, and their experimental data have been published earlier [21–23]. Coumarin derivatives that were used in the experiment were 3-(4-trifluoromethoxyphenyl)-6-methoxycoumarin, 3-(3-benzyloxo)phenyl-7-methoxycoumarin, 3-(3-fluoro-4-hydroxyphenyl)-6-methoxycoumarin, 3-(3-methoxyphenyl)coumarin, and 7-hydroxycoumarin derivatives 3-(1H-1, 2, 4-triazol-1-yl)-7-hydroxycoumarin and 3-(4-methyl-phenyl)-7-hydroxycoumarin.

### 2.2. Mice

Three-months-old male LDLr^−/−^ApoB^100/100^ mice from the colony of the Center of Experimental Animals at the University of Eastern Finland (129sv/B6 mixed background, backcrossed ten times, The Jackson Laboratory, Bar Harbor, ME, USA) were randomly divided into two groups, a Western diet (WD) (*n* = 10) and regular chow diet (*n* = 8) groups. The WD group was fed with the WD (Harlan Teklad 88137, containing 42% of kcal from fat), and control mice received a standard diet (Teklad Global 16% protein rodent diet: 12% of calories from fat and 0% cholesterol) for five months. Mice were euthanized with CO_2_ and perfused with phosphate-buffered saline to collect liver samples for histology or snap frozen in liquid nitrogen. Mice were housed in groups with free access to tap water in a room with a controlled 55% humidity and a temperature of 22°C. A 12 h light and 12 h dark environmental light cycle was maintained.

The experimental design was approved by the Animal Ethics Committee of the State Provincial Office of Southern Finland (Decision number ESAVI/11642/04.10.07/2014). All the experiments conform to the guidelines from Directive 2010/63/EU of the European Parliament on the protection of animals used for scientific purposes and by the ARRIVE guidelines.

### 2.3. Histology

Collected liver samples were fixed in 4% paraformaldehyde in PBS (pH 7.4) overnight and then embedded in paraffin. Tissue sections were cut (5 *μ*m) and stained with hematoxylin-eosin to evaluate hepatic steatosis and fibrosis. The histological diagnosis of steatosis was classified into four categories: (0) normal liver with simple steatosis (steatosis <5%), (1) mild steatosis (steatosis 5–33%), (2) moderate steatosis (steatosis 33–66%), and (3) advanced steatosis (>66%) according to standard histopathological criteria [24, 25]. To evaluate fibrosis, serial sections were stained with Masson's Trichrome. All images were analyzed in a blinded manner with a color image analysis system based on a threshold (Image *J* 1.48 V Software).

### 2.4. Preparation of Microsomes

Cytosol and microsome specimens were prepared from frozen livers as described earlier [26]. Shortly, the liver tissue samples (left and right lobes of the liver) were randomized, weighed, gently thawed, and homogenized with Potter–Elvehjem homogenizer in 100 mM Tris-HCl buffer pH 7.4 containing 1 mM EDTA (4 parts buffer to 1 part tissue). The homogenate was centrifuged at 10 000 g for 15 min at 4°C. The supernatant was collected and centrifuged at 100 000 g for 60 min at 4°C. The supernatant and pellet were separated. The pelleted microsomal fraction was rehomogenized with Potter–Elvehjem homogenizer in 100 mM Tris-HCl buffer, pH 7.4, containing 0.1 mM EDTA and 20% glycerol. The protein concentration was determined with the Bradford method of Bio-Rad protein. The supernatant and microsomal samples were stored at −80°C until further use.

### 2.5. CYP Enzyme Activity Assays

CYP oxidation reactions took place in 100 *μ*L volume containing 100 mM Tris-HCl pH 7.4, 20% NADPH regenerating system, liver microsomes as the enzyme source, and substrates of 1 *μ*M 7-ethoxyresorufin, 1 *μ*M 7-pentoxyresorufin, 10 *μ*M coumarin, 10 *μ*M 3-(4-trifluoromethoxyphenyl)-6-methoxycoumarin, 10 *μ*M 3-(3-benzyloxo)phenyl-7-methoxycoumarin, 10 *μ*M 3-(3-fluoro-4-hydroxyphenyl)-6-methoxycoumarin, or 10 *μ*M 3-(3-methoxyphenyl)coumarin. Blank samples did not contain liver microsomes.

Measurements (both CYP and conjugation) were carried out in a randomized order of samples at 37°C in a 96 multiwell plate format following fluorescence every other minute for 40 min using an excitation filter at 405 nm and detection at 460 nm for oxidation of coumarin and coumarin derivatives, and at excitation 570 nm and emission 615 nm for 7-ethoxyresorufin or 7-pentoxyresorufinO-dealkylations in a Victor^2^ 1420 Multilabel counter (PerkinElmer, Life Sciences, Turku, Finland). Resorufin was used as a standard for 7-O-dealkylation of resorufins and 7-hydroxycoumarin as the surrogate standards for oxidation of coumarin or coumarin derivatives to calculate the amount of product formed. The linear phase of the reactions was used for calculations of CYP activities.

7-Ethoxyresorufin, coumarin, and 7-pentoxyresorufin are classical probe substrates of CYP1, CYP2A, and CYP2B enzymes, respectively, in animals including mice [27, 28]. 3-(4-trifluoromethoxyphenyl)-6-methoxycoumarin, 3-(3-methoxyphenyl)coumarin, and 3-(3-fluoro-4-hydroxyphenyl)-6-methoxycoumarin are new CYP probe substrates [23], which have selectivity for human CYP1 and which have not been tested earlier with mice samples; 3-(3-benzyloxo)phenyl-7-methoxycoumarin is a new CYP probe substrate, which is oxidized by several human CYPs such as CYP1, CYP2D, CYP3A, and CYP2C and which has not been tested earlier with mice samples. Because their corresponding human CYPs are generally recognized as important drug oxidizing enzymes; they were chosen for this study.

### 2.6. Conjugations

The incubation assays of the mixture of glucuronidation were done in 100 *μ*L volume containing 100 mM phosphate buffer pH 7.4, 5 mM MgCl_2_, 0.25 mg/L alamethicin, liver microsomes, 0.5 mM uridine diphosphate glucuronic acid (UDPGA), and 10 *μ*M 7-hydroxy-4-trifluoromethylcoumarin (HFC) or 3-(1H-1, 2, 4-triazol-1-yl)-7-hydroxycoumarin. The glucuronidation blank samples did not contain microsomes. The incubation mixture of sulfonation assays were done in 100 *μ*L containing 100 mM phosphate buffer pH 7.4, 5 mM MgCl_2_, 100 *μ*M PAPS, mouse liver cytosol, and 10 *μ*M 3-(4-methylphenyl)-7-hydroxycoumarin or HFC. The sulfonation blank samples did not contain cytosol. The incubation mixture of esculetin COMT reactions were done in a 100 *μ*L volume containing 100 mM phosphate buffer pH 7.4, 5 mM MgCl_2_, 100 *μ*M S-adenosylmethionine (SAM), 10 *μ*M esculetin, and microsomes or cytosol as the enzyme source. The blank samples did not contain either microsomes or cytosol. Measurements were carried out at 37°C in a 96 multiwell plate format following fluorescence every other minute for 40 min using an excitation filter at 405 nm and detection at 460 nm in a Victor^2^ 1420 Multilabel counter (PerkinElmer, Life Sciences, Turku, Finland). Substrates acted themselves as standards and at every time point was calculated its own standard line for quantitation before plotting concentrations against the incubation time. The linear phase of the reactions was used for calculations of the conjugation rate.

Glucuronidation of HFC is catalyzed by several human UGTs such as UGT1A6, A7, A9, A10, 2A1, and 2B7, and 3-(1H-1, 2, 4-triazol-1-yl)-7-hydroxycoumarin by human UGT1A10, but their UGT selectivity in mice is not known [29, 30]. Sulfonation selectivity of 3-(4-methylphenyl)-7-hydroxycoumarin or HFC by SULT enzymes is not known at the moment.

### 2.7. Statistical Analysis

An unpaired parametric *t*-test with two-sided*p* values was carried out on data of all 14 enzyme activities using GraphPad Prism 5 software. For multivariate analysis, SIMCA 15.0. Umetrics software was used when conducting the principal component analysis (PCA) to investigate latent components explaining the variation in the data and partial least squares discriminant analysis (PLS-DA) to determine latent components explaining the separation between the study groups. Numerical values are shown as geometrical mean ± 95% confidence interval (CI).

## 3. Results

### 3.1. Prolonged WD Induces Hepatic Steatosis and Fibrosis in Male LDLr^−/−^ApoB^100/100^ Mice

**Figure 1:**
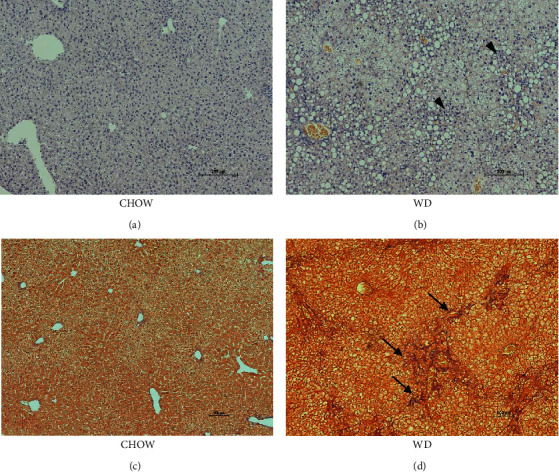
Prolonged Western diet (WD) induces hepatic steatosis in male LDLr^−/−^ApoB^100/100^ mice. LDLr^−/−^ApoB^100/100^ male mice were fed the WD for 5 months. When detecting hepatic steatosis, liver sections were stained with hematoxylin-eosin (a) on a chow diet and (b) after 5 months on the WD. When determining liver fibrosis, liver sections (c) on a chow and (d) after the WD was stained with Masson trichrome stain that designates fibrosis with blue color (arrow). The WD induced high increase in hepatic lipid accumulation and infiltration of inflammatory cells (arrowhead) and promoted liver fibrosis. Representative pictures of livers are shown with a scale bar 200 *μ*m.

To induce hepatic steatosis, male LDLr^−/−^ApoB^100/100^ mice were fed with the WD for five months, whereas the control mice received the standard chow diet. Before the diet, the body weight was on average 20 g. After the chow diet, average weight was 30 g, and after the WD, average weight was 40 g. The WD induced a clear increase in hepatic lipid accumulation, but on a chow diet, hepatic steatosis was not detected (Figures 1(a) and 1(b)). In addition, the WD increased the infiltration of inflammatory cells, such as neutrophils and lymphocytes, and promoted the formation of early fibrosis (Figure 1(d)), which was not detected on a chow diet in male LDLr^−/−^ApoB^100/100^ mice after 5 months (Figure 1(c)).

### 3.2. The Effect of WD on Liver Drug Metabolic Activities

**Figure 2:**
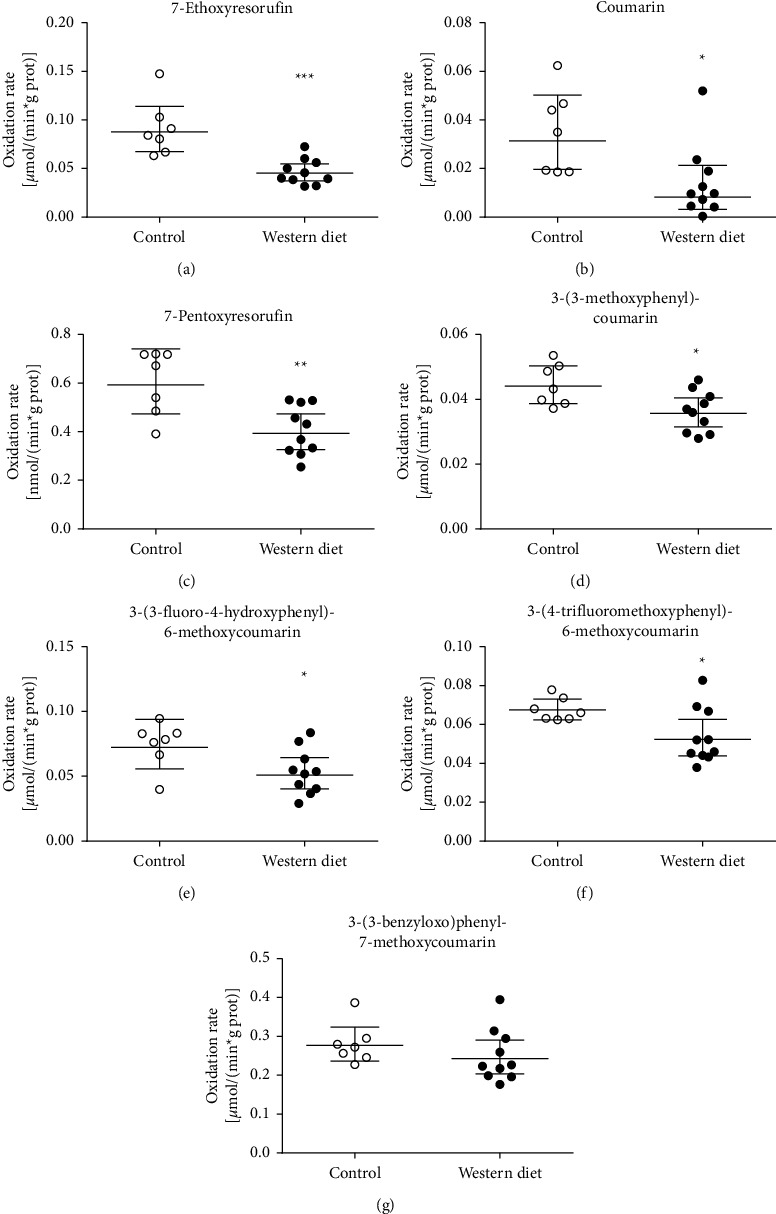
The effect of consumption of the Western diet (WD) on the probe protein-normalized activities of CYP enzymes in the liver microsomes of male LDLr^−/−^ApoB^100/100^ mice. The liver microsomes were prepared from the livers of eight months old mice; the animals were fed either with the WD or the chow diet for five months. CYP-oxidized reactions were determined with the indicated probe substrates: 7-ethoxyresorufin, 7-pentoxyresorufin, coumarin, 3-(4-trifluoromethoxyphenyl)-6-methoxycoumarin, 3-(3-benzyloxo)phenyl-7-methoxycoumarin, 3(3-fluoro-4-hydroxyphenyl)-6-methoxycoumarin, and 3-(3-methoxyphenyl)coumarin. ^*∗*^, *p* < 0.05; ^*∗∗*^, *p* < 0.01; and ^*∗∗∗*^, *p* < 0.001 WD vs. chow diet. The lines of the groups represent geometrical means with 95% confidence interval: (a) 7-ethoxyresorufin, (b) coumarin, (c) 7-pentoxyresorufin, (d) 3-(3-methoxyphenyl)-coumarin, (e) 3-(3-fluoro-4-hydroxyphenyl)-6-methoxycoumarin, (f) 3-(4-trifluoromethoxyphenyl)-6-methoxycoumarin, and (g) 3-(3-benzyloxo)phenyl-7-methoxycoumarin.

**Figure 3:**
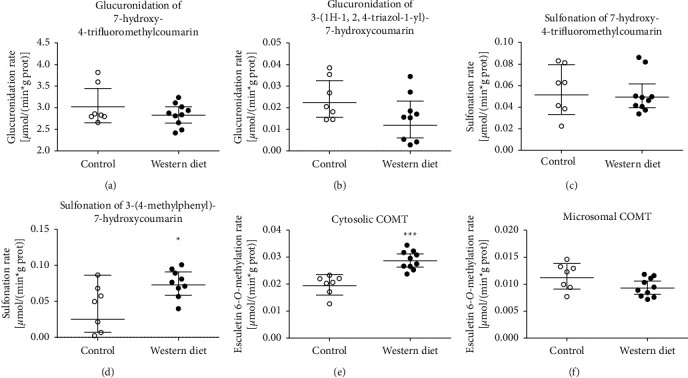
The effect of the Western diet (WD) on the probe activities of conjugation enzymes in the liver cytosol or microsomes of male LDLr^−/−^ApoB^100/100^ mice. The liver microsome or cytosol samples were prepared from livers of eight months old mice; the animals were fed either with the WD or the chow diet for five months. Glucuronidation, sulfonation, and catechol (O)-methylation (COMT) activities determined with the indicated probe substrates. ^*∗*^, *p* <0.05 ^*∗∗∗*^, *p* < 0.001 WD vs. chow diet. The lines of the groups represent geometrical means with 95% confidence interval: (a) glucuronidation of 7-hydroxy-4-trifluoromethylcoumarin, (b) glucuronidation of 3-(1H-1,2,4-triazol-1-yl)7-hydroxycoumarin, (c) sulfonation of 7-hydorxy-4-trifluoromethylcoumarin. (d) sulfonation of 3-(4-methylphenyl)-7-hydroxycoumarin, (e) cytosolic COMT, and (f) Microsomal COMT.

Seven CYP, two UGT, two SULT, and two COMT probe activities were measured from liver microsomes or cytosol of both chow diet and WD male LDLr^−/−^ApoB^100/100^ mice. The activity was calculated per amount of protein and per liver, as accumulation of fat could affect protein concentration and liver size. Consequently, activity per liver indicated total liver metabolic capacity. The consumption of the WD for five months decreased significantly six microsomal CYP probe activities and had no significant effect on the 3-(3-benzyloxo)phenyl-7-methoxycoumarin 7-*O*-demethylation rate in both the protein-normalized and per liver activity analysis (Figure 2 and Table 1). In protein-normalized analysis, the WD decreased most extensively in the coumarin 7-hydroxylation activity by decreasing it to 26% from the rate of chow diet mice. Other protein-normalized activities were decreased less, varying from 52% (7-ethoxyresorufin 7-O-deethylation) to 81% (7-hydroxylation of 3-(3-methoxyphenyl)coumarin) compared to the rates of chow diet mice. In the rates of glucuronidation, sulfonation, and microsomal COMT, there were no differences in the protein-normalized activities between samples of the chow diet and WD (Figure 3), but activities of both glucuronidation and sulfonation of 4-trifluoromethyl-7-hydroxycoumarin and microsomal COMT per liver were decreased significantly (Table 1). Protein-normalized cytosolic COMT activity was 48% higher in WD mice compared to chow diet mice (Figure 3), but the per liver activity difference was not statistically significant (Table 1). In male LDLr^−/−^ApoB^100/100^ mice, the WD affected more significantly on CYP probe activities than conjugation probe activities. Primarily, the WD decreased the CYP enzyme probe activities.

**Figure 4:**
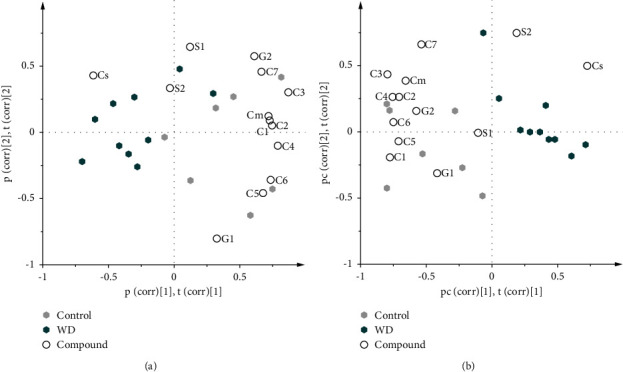
Multivariate analysis separated the Western diet (WD) fed male LDLr^−/−^ApoB^100/100^ mice into low xenobiotic metabolism activity group in contrast to mice fed with the chow diet. Biplots showing the first two latent components of the principal component analysis ((a) cumulative *R*^2^*X* = 0.99 and cumulative *Q*^2^ = 0.58) and a partial least sum of squares discriminant analysis ((b) cumulative *R*^2^*Y* = 0.86 and cumulative *Q*^2^ = 0.66) of CYP oxidation, glucuronidation, sulfonation, and COMT activities in liver of control and WD male LDLr^−/−^ ApoB^100/100^ mice. Explanations of the symbols: C1 = ethoxyresorufin 7-(O)-deethylation, C2 = 7-hydroxylation of 3-(4-trifluoromethoxyphenyl)-6-methoxycoumarin, C3 = 7-hydroxylation of 3-(3-fluoro-4-hydroxyphenyl)-6-methoxycoumarin, C4 = coumarin 7-hydroxylation, C5 = pentoxyresorufin 7-(O)-depentylation, C6 = 7-hydroxylation of 3-(3-methoxyphenyl)coumarin, C7 = 7-(O)-demethylation of 3-(3-benzyloxo)phenyl-7-methoxycoumarin, G1 = HFC glucuronidation, G2 = 3-(1H-1, 2, 4-triazol-1-yl)-7-hydroxycoumarin glucuronidation, S1 = HFC sulfonation, S2 = 3-(4-methylphenyl)-7-hydroxycoumarin sulfonation, Cs = cytosol COMT, and Cm = microsomal COMT.

All CYP oxidation and conjugation data were analyzed by PCA (cumulative *R*^2^*X* = 0.99, cumulative *Q*^2^ = 0.58) and PLS-DA (cumulative *R*^2^*Y* = 0.857, cumulative *Q*^2^ = 0.655) models (Figure 4). Both models showed a separation of the study groups. The models separated male LDLr^−/−^ApoB^100/100^ mice according to diets of chow and WD groups (Figure 4). In the PLS-DA model, higher cytosolic COMT and lower CYP oxidations and glucuronidation of substrates were associated with the WD male LDLr^−/−^ApoB^100/100^ mice. Moreover, oxidation of 3-(3-benzyloxo)phenyl-7-methoxycoumarin (C7) and sulfonation of 3-(4-methylphenyl)-7-hydroxycoumarin (S2) separated individual male LDLr^−/−^ApoB^100/100^ mice.

### 3.3. The Correlation of CYP, UGT, SULT, or COMT Activities

**Figure 5:**
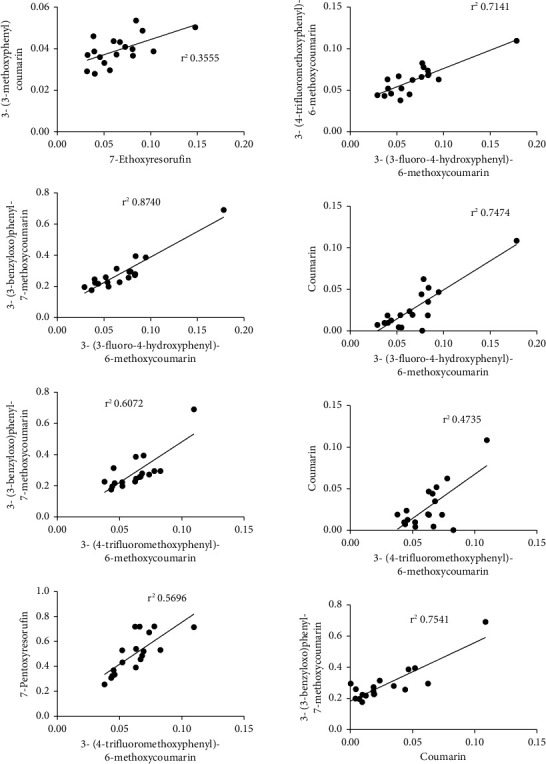
Statistically significant linear correlations between the oxidation rates of two probe substrates in liver microsomes in Figure 2 and Table 2.

There were eight significant linear correlations found among the oxidation rates of seven probe CYP substrates (Figure 5, Table 2). Four activities correlated with each other: 7-O-demethylation of 3-(3-fluoro-4-hydroxyphenyl)-6-methoxycoumarin (C3) and 3-(3-benzyloxo)phenyl-7-methoxycoumarin, and 7-hydroxylation of coumarin (C4) and 3-(4-trifluoromethoxyphenyl)-6-methoxycoumarin (C2) pointing six linear correlations. Two other linear correlations were noted between 7-O-deethylation of 7-ethoxyresorufin (C1) and 7-hydroxylation of 3-(3-methoxyphenyl)coumarin (C6), and between 7-hydroxylation of 3-(4-trifluoromethoxyphenyl)-6-methoxycoumarin (C2) and 7-O-depentylation of 7-pentoxyresorufin (C5). The highest statistical correlation was observed between rates of 7-hydroxylation of 3-(3-fluoro-4-hydroxyphenyl)-6-methoxycoumarin and 7-O-demethylation of 3-(3-benzyloxo)phenyl-7-methoxycoumarin (*r*^2^ 0.874). The rate of 7-hydroxylation of 3-(3-fluoro-4-hydroxyphenyl)-6-methoxycoumarin correlated also strongly with coumarin 7-hydroxylation (*r*^2^ 0.7474) and 7-hydroxylation of 3-(4-trifluoromethoxyphenyl)-6-methoxycoumarin rates (*r*^2^ 0.7141). The 7-pentoxyresorufin 7-O-depentylation rate correlated with the 7-hydroxylation of 3-(4-trifluoromethoxyphenyl)-6-methoxycoumarin rate (*r*^2^ 0.5696). The 7-ethoxyresorufin 7-O-deethylation rate correlated weakly with the 7-hydroxylation of 3-(3-methoxyphenyl)coumarin rate (*r*^2^ 0.3555). The activities of two glucuronidation, two sulfonation, and one microsomal COMT, and one cytosol COMT enzymes did not correlate among each other. Correlation among oxidation rates of CYP probe substrates mean that the same CYP enzyme is significantly involved in the oxidation of the correlated probe substrates. The lack of a significant correlation between two sulfonation or glucuronidation substrates means that two different conjugating enzymes are involved in their catalysis.

## 4. Discussion

The liver is an important organ for kinetics of drugs and other xenobiotics because all compounds absorbed from the gastrointestinal tract have to pass through the liver before gaining access to the systematic circulation and further distribution in the body. Secondly, of all organs, the liver has the highest ability and capability to metabolize xenobiotics [4, 5]. High-fat diets, such as WD, alters the function of the liver and affects hepatic xenobiotic metabolism [31]. In this study, the effects of the WD feeding on hepatic xenobiotic metabolism were studied in genetically modified hypercholesterolemic LDLr^−/−^ApoB^100/100^ male mice. Prolonged WD consumption induced severe lipid accumulation, chronic inflammation, and early fibrosis in male LDLr^−/−^ApoB^100/100^ mice liver. Mice fed with a WD had lower hepatic activities of several drug-metabolizing enzymes than mice fed with the chow diet. The declines of CYP enzyme activities were statistically significant, whereas the decreases in conjugation activities of glucuronidation and sulfonation were not statistically significant. The chow diet and WD mice were observed to differ metabolically, as multivariate PLC-DA analysis separated them to higher metabolism control and lower metabolism WD groups. According to these results, WD-induced hepatic steatosis decreased the overall liver xenobiotic metabolism of male LDLr^−/−^ ApoB^100/100^ mice.

The effects of WD on the oxidation rates of seven probe substrates of CYP enzymes were determined in this study. Ethoxyresorufin 7-O-deethylation [32], coumarin 7-hydroxylation [28], and pentoxyresorufin 7-O-depentylation [33] are identified as the probe substrates of CYP1, CYP2A, and CYP2B enzymes in mice, respectively. Four coumarin derivatives of this study are new, convenient profluorescent probe substrates of CYP enzymes. 7-hydroxylations of 3-(4-trifluoromethoxyphenyl)-6-methoxycoumarin, 3-(3-methoxyphenyl)coumarin, and 3-(3-fluoro-4-hydroxyphenyl)-6-methoxycoumarin are catalyzed efficiently by human CYP1 enzymes [23]. However, only the 7-hydroxylation of 3-(3-methoxyphenyl)coumarin correlated linearly significantly with ethoxyresorufin 7-O-deethylation. Two other coumarin derivatives did not correlate with ethoxyresorufin 7-O-deethylation, but they correlated with coumarin 7-hydroxylation. Therefore, the oxidation ability of CYP2A rather than CYP1 enzymes in the mouse liver were measured with 3-(4-trifluoromethoxyphenyl)-6-methoxycoumarin and 3-(3-fluoro-4-hydroxyphenyl)-6-methoxycoumarin. In addition, 7-O-demethylation of 3-(3-benzyloxo)phenyl-7-methoxycoumarin correlated linearly and significantly with coumarin 7-hydroxylation. Therefore, these four substrates seem to be oxidized mainly by mouse CYP2A enzymes and to a lesser extent by some other CYP enzyme, which differs from the previous substrate selectivity results among the human CYP enzymes [23]. Pentoxyresorufin, the 7-O-depentylation probe substrate of CYP2B, did not linearly correlate with the oxidation rates of the other six substrates. It can be concluded from the linear correlation results that firstly the CYP enzyme selectivity of coumarin derivatives differ between humans and mice, and secondly, the CYP probe substrates used in this study mainly measured the effects of WD on CYP1, CYP2A, and CYP2B enzymes in hypercholesterolemia-inducing transgenic LDLr^−/−^ApoB^100/100^ male mice. It is concluded that the decrease of drug metabolism was due to a decrease in the activity of CYP1, CYP2A, and CYP2B in the liver.

In contrast, high-fat diets have been observed to increase liver CYP activities in several animal models. In A/J and wild-type mice, a high-fat diet increased both hepatic CYP1A2 [34] and 2E1 activity, respectively, compared to a low-fat diet [35]. CYP2E1 is involved in the metabolism of ketogenic substances [36]. Furthermore, a high-fat diet has increased significantly CYP2A5 enzyme activity in C57BL/6J mice [37]. A high-fat diet has been shown to significantly increase CYP1A2, 2E1, 2C, and 4A enzyme activities in streptozotocin-induced diabetes in Sprague–Dawley rats [38]. In male domestic pigs, the continuous consumption of the high-fat diet increased the CYP2E1 activity but did not alter 1A or 2B enzyme activity [39]. In C57BL/6 mice, the WD has not altered liver mRNA levels of xenobiotic metabolizing CYPs, but instead has decreased mRNA levels of CYP7 and CYP51 [40], which are bile acid and steroid synthetizing CYPs, respectively. Single- or seven-day daily i.p. exposure of oil has reduced the amounts of CYP enzyme proteins and activities in the mice liver [41]. Alternatively, in rats, the lipid exposure has increased the hepatic activity and protein level of CYP2E [42, 43]. In Sprague–Dawley rats, a high-fat diet has exerted no significant effect on CYP2B1 mRNA or protein expression levels [44]. In two of these studies, the high-fat diets have resulted in hepatic steatosis and inflammation in C57BL/6J mice liver histology [35, 37]. In summary, it is evident that the effect of consuming high-fat diet on liver CYP enzyme activities is dependent on the duration and amount of fat in diet and severity of the effect on the liver histology.

Our study has some limitations. We did not monitor consumption of the food or caloric intake. The weight of the mice was measured only before and after WD. We did not include wild-type mice in this study. However, LDLr^−/−^ApoB^100/100^ mice is a model of human familial hypercholesterolemia, so these results may have implications in individuals with hypercholesterolemia. In addition, the experiments were performed only on male mice, which may affect the generalizability of the results.

In our study, WD in male LDLr^−/−^ApoB^100/100^ mice did not have any significant effects on conjugation reactions of UGT, SULTs, and cytosolic COMT. There are a few studies examining the effects of high-fat diet on UGT and membrane-bound COMT expression levels. In both male and female type 2 diabetes-induced Sprague–Dawley rats treated with streptozotocin, consumption of a high-fat diet increased UGT1A1, UGT1A6, and UGT1A7 probe activities but decreased the UGT2B1 probe activity [45]. The relative expression of membrane-bound COMT protein was decreased significantly in mice [46]. There was no data found on the effects of a high-fat diet on SULTs or cytosolic COMT.

## 5. Conclusions

The effects of WD on the liver drug metabolism in male LDLr^−/−^ApoB^100/100^ mice were studied here for the first time. The five-month consumption of the WD-induced hepatic steatosis in male LDLr^−/−^ApoB^100/100^ mice. CYP1, CYP2A, and CYP2B probe activities were significantly lower in mice fed with the WD than in mice who were fed with a standard chow diet. No clear effects were observed on conjugation reactions of glucuronidation, sulfonation, and catechol O-methylation after feeding with a WD. Multivariate analysis from hepatic drug metabolizing probe activities separated WD mice into a low xenobiotic metabolism activity group and the chow diet mice as the high xenobiotic metabolism activity group. In conclusion, WD-induced obesity and hepatic steatosis were associated with decreased liver xenobiotic metabolism in male LDLr^−/−^ApoB^100/100^ mice.

## Figures and Tables

**Table 1:** The effect of a Western diet (WD) on the total probe drug metabolism of the mouse model of NAFLD. The liver microsome and cytosol fractions were prepared from the livers of eight months old male LDLr^−/−^ ApoB^100/100^ mice that were fed either with chow (8 mice) or WD (10 mice) for five months.

Drug metabolizing activity	nmol/(min *∗* liver) mean (95% confidence limit)	
	Control diet	Western diet
Ethoxyresorufin 7-O-deethylation	2.2 (1.8–2.6)	1.0 (0.6–1.4)^*∗∗∗*^
Coumarin 7-hydroxylation	1.0 (0.4–1.6)	0.30 (0.08–0.52)^*∗∗*^
Pentoxyresorufin 7-O-depentylation	0.018 (0.011–0.024)	0.0083 (0.0054–0.011)^*∗∗*^
7-hydroxylation of 3-(3-methoxyphenyl)coumarin	1.3 (0.9–1.7)	0.71 (0.54–0.88)^*∗∗*^
7-hydroxylation of 3-(3-fluoro-4-hydroxyphenyl)-6-methoxycoumarin	2.1 (1.4–2.8)	1.2 (0.7–1.7)^*∗*^
7-hydroxylation of 3-(4-trifluoromethoxyphenyl)-6-methoxycoumarin	1.7 (1.3–2.1)	1.0 (0.6–1.4)^*∗*^
7-O-demethylation of 3-(3-benzyloxo)phenyl-7-methoxycoumarin	7.8 (5.9–9.8)	5.4 (3.2–7.5)
Glucuronidation of 4-trifluoromethyl-7-hydroxycoumarin	88 (54–121)	57 (42–73)^*∗*^
Glucuronidation of 3-(1H-1, 2, 4-triazol-1-yl)-7-hydroxycoumarin	0.67 (0.41–0.92)	0.35 (0.10–0.61)
Sulfonation of 4-trifluoromethyl-7-hydroxycoumarin	11 (7.5–14)	5.9 (4.0–7.8)^*∗∗*^
Sulfonation of 3-(4-methylphenyl)-7-hydroxycoumarin	8.4 (2.4–14)	8.8 (5.5–12)
Cytosolic COMT	3.9 (3.3–4.4)	3.3 (2.7–4.0)
Microsomal COMT	0.64 (0.47–0.82)	0.38 (0.27–0.49)^*∗∗*^

*P* values: ^*∗*^ < 0.05; ^*∗∗*^ < 0.01; ^*∗∗∗*^ < 0.001 Western diet vs. chow diet.

**Table 2:** Linear correlation coefficients between oxidation rates of two CYP substrates or between rates of two conjugation substrates and their respective *P* values in liver samples of male LDLr^−/−^ApoB^100/100^ mice fed with the chow or Western diet. The rates of CYP oxidation, glucuronidation, sulfonation, or COMT with the indicated substrates were determined as described in Materials and Methods. Correlations were calculated from the data in Figures 2 and 3.

Oxidation substrate 1-oxidationsubstrate 2	Correlation coefficient *r*^2^	*P* value
7-ethoxyresorufin (C1)-3-(3-fluoro-4-hydroxyphenyl)-6-methoxycoumarin (C3)	0.1597	0.1003
7-ethoxyresorufin (C1)-3-(4-trifluoromethoxyphenyl)-6-methoxycoumarin (C2)	0.1265	0.1475
**7-ethoxyresorufin** (C1)-**3-(3-methoxyphenyl)coumarin (C6)**	**0.3555**	**0.0090**
7-ethoxyresorufin (C1)-3-(3-benzyloxo)phenyl-7-methoxycoumarin (C7)	0.07485	0.2720
7-ethoxyresorufin (C1)-coumarin (C4)	0.1783	0.0809
7-ethoxyresorufin (C1)-7-pentoxyresorufin (C5)	0.07366	0.2760
**3-(3-fluoro-4-hydroxyphenyl)-6-methoxycoumarin (C3)-3-(4-trifluoromethoxyphenyl)-6-methoxycoumarin (C2)**	**0.7141**	**<0.0001**
3-(3-fluoro-4-hydroxyphenyl)-6-methoxycoumarin (C3)-3-(3-methoxyphenyl)coumarin (C6)	0.02517	0.5295
**3-(3-fluoro-4-hydroxyphenyl)-6-methoxycoumarin (C3)-3-(3-benzyloxo)phenyl-7-methoxycoumarin(C7)**	**0.8740**	**<0.0001**
**3-(3-fluoro-4-hydroxyphenyl)-6-methoxycoumarin (C3)-coumarin (C4)**	**0.7474**	**<0.0001**
3-(3-fluoro-4-hydroxyphenyl)-6-methoxycoumarin (C3)-7-pentoxyresorufin (C5)	0.2930	0.0203
3-(4-trifluoromethoxyphenyl)-6-methoxycoumarin (C2)-3-(3-methoxyphenyl)coumarin (C6)	0.04947	0.3750
**3-(4-trifluoromethoxyphenyl)-6-methoxycoumarin (C2)-3-(3-benzyloxo)phenyl-7-methoxycoumarin(C7)**	**0.6072**	**0.0001**
**3-(4-trifluoromethoxyphenyl)-6-methoxycoumarin (C2)-coumarin (C4)**	**0.4735**	**0.0016**
**3-(4-trifluoromethoxyphenyl)-6-methoxycoumarin (C2)-7-pentoxyresorufin (C5)**	**0.5685**	**0.0003**
3-(3-methoxyphenyl)coumarin (C6)-3-(3-benzyloxo)phenyl-7-methoxycoumarin **(C7)**	0.004639	0.7883
3-(3-methoxyphenyl)coumarin (C6)-coumarin (C4)	0.1546	0.1065
3-(3-methoxyphenyl)coumarin (C6)-7-pentoxyresorufin (C5)	0.2151	0.0526
**Coumarin (C4)-3-(3-benzyloxo)phenyl-7-methoxycoumarin (C7)**	**0.7541**	**<0.0001**
Coumarin (C4)-7-pentoxyresorufin (C5)	0.2597	0.0308
3-(3-benzyloxo)phenyl-7-methoxycoumarin (C7)-7-pentoxyresorufin (C5)	0.1527	0.1089
**Conjugation substrate 1-conjugation substrate 2**		
Glucuronidation 7-hydroxy-4-trifluoromethylcoumarin (G1)-3-(1H-1, 2, 4-triazol-1-yl)-7-hydroxycoumarin (G2)	0.03156	0.4807
Sulfonation 7-hydroxy-4-trifluoromethylcoumarin (S1)-3-(4-methylphenyl)-7-hydroxycoumarin (S2)	0.0006785	0.9183
COMT cytosol (Cc)-COMT microsomes (Cm)	0.06438	0.3096

Values in bold indicate a statistically significant correlation. Letter number abbreviations, e.g., C1 in the brackets after the substrate point to the reactions of Figure 4. Values in bold indicate a statistically significant correlation. Letter number abbreviations, e.g., C1 in the brackets after the substrate point to the reactions in Figure 4.

## Data Availability

The analyzed data used to support the findings of this study are included within the article.
